# Correction to ‘Mutagenesis of the DNA binding residues in bovine pancreatic DNase1: an investigation into the mechanism of sequence discrimination by a sequence selective nuclease’

**DOI:** 10.1093/nar/gkad039

**Published:** 2023-01-18

*Nucleic Acids Research*, Volume 19, Issue 22, 25 November 1991, Pages 6129–6132, https://doi.org/10.1093/nar/19.22.6129

In the HTML version of this manuscript, Andrew F. Worrall's surname was misspelled.

The Publisher apologizes for this error, which has now been corrected.

